# Off-pump bi-directional Glenn shunt: How I do it?

**DOI:** 10.4103/0974-2069.43879

**Published:** 2008

**Authors:** Anil Bhan

**Affiliations:** Department of Cardiovascular and Thoracic Surgery, Batra Hospital and Medical Research Centre, New Delhi, India

**Keywords:** Bidirectional Glenn shunt, cavopulmonary shunt, off pump surgery

## INTRODUCTION

The bi-directorial cavopulmonary (Glenn) shunt (BDG) was introduced in 1972 by Azzolina *et al*[1] and is a commonly performed procedure for a variety of cyanotic congenital heart diseases that eventually lead to a single ventricle repair.[2] Most of the surgeons prefer to perform this procedure under cardiopulmonary bypass (CPB) with its associated detrimental effects and the added cost. The performance of this operation without CPB is associated with significant elevation of the proximal superior vena cava (SVC) pressure that may lead to neurological injury. The technique of doing BDG without CPB has been reported by many authors.[3–10] However most of these reports have recommended some means of decompressing the proximal SVC at the time of clamping it. Our group has been doing this procedure on selected patients without any CPB and without using any decompressive techniques for the SVC.

## CLINICAL MATERIAL

Between January 2002 and August 2008, 37 patients have undergone BDG operation by the author at three institutions viz. All India Institue of Medical Sciences, Max Devki Devi Heart and Vascular Institute and Batra Hospital and Medical Research Centre, New Delhi, India

### Pre-operative evaluation

We prefer to perform this procedure in children from nine months to 1 year of age, although smaller children and even adults can safely undergo this operation. The feasibility of doing the off pump BDG shunt was determined more by the size of superior vena cava and pulmonary arteries rather than the age of the patient.

The patients were evaluated by pre-operative echocardiography and cardiac catheterization. The size of the atrial septal defect and atrio-ventricular valve was assessed. Need for any intra cardiac procedure and low baseline preoperative oxygenation mandated the exclusion of patients from this group. Patients with functioning right or left Blalock-Taussig (BT) shunt were good candidates for the procedure, as most of these shunts were done from thoracotomy and an adequate intrapericardial length of pulmonary artery was available for the BDG shunt. Pre-procedure oxygen saturation in such patients was almost always > 75 % and therefore was never an issue. Prior pulmonary artery banding was not a contraindication to this operation.

### Operative technique

**Figure 1:**
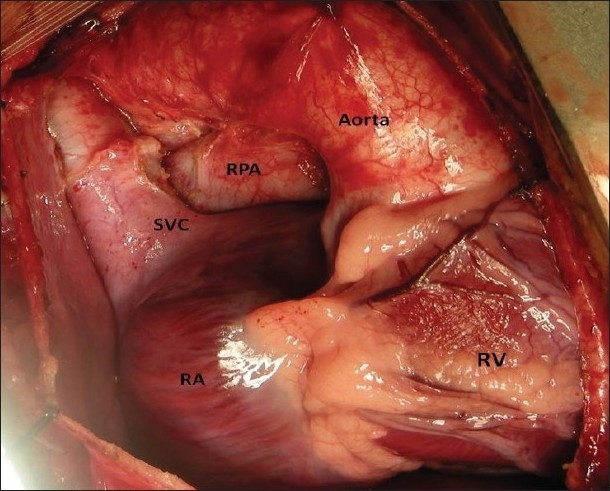
A good exposure of the superior vena cava and the right pulmonary aretry is essential

**Figure 2:**
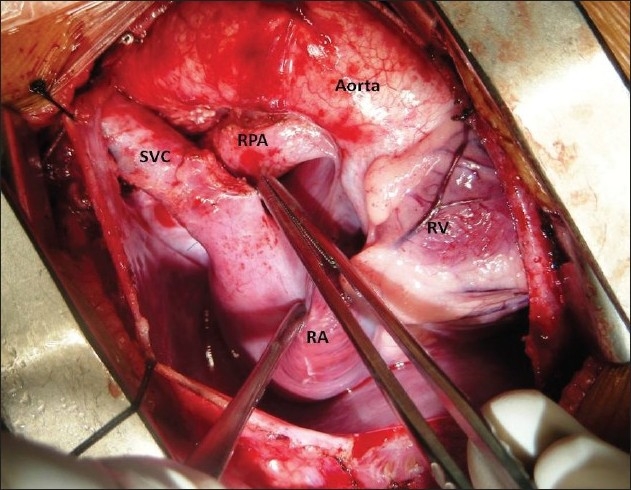
Superior vena cava is completely skeletonized

**Figure 3:**
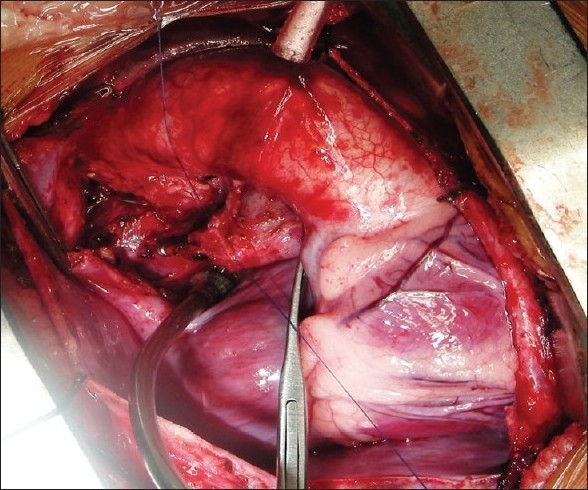
The right pulmonary artery is dissected and an adequate length exposed

**Figure 4:**
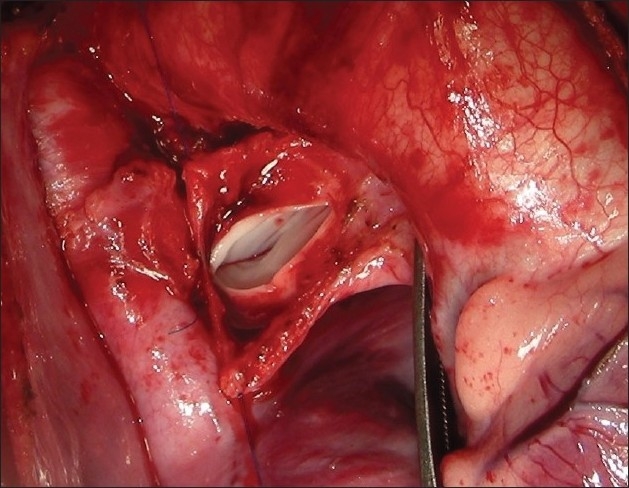
The right pulmonary artery is opened between two stay sutures with adequate proximal and distal control

**Figure 5:**
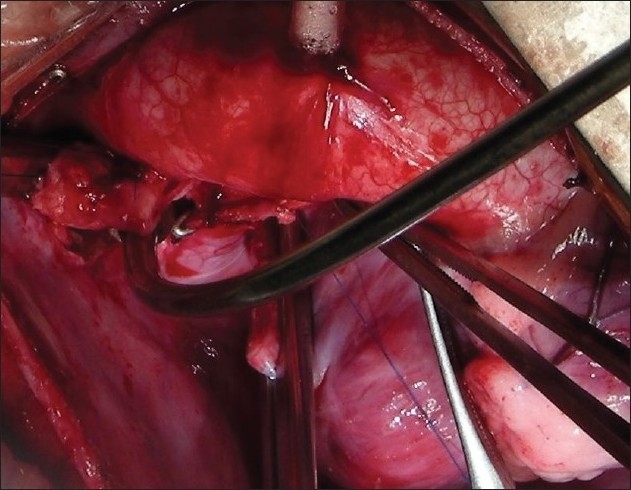
The superior vena cava is transected between clamps

**Figure 6:**
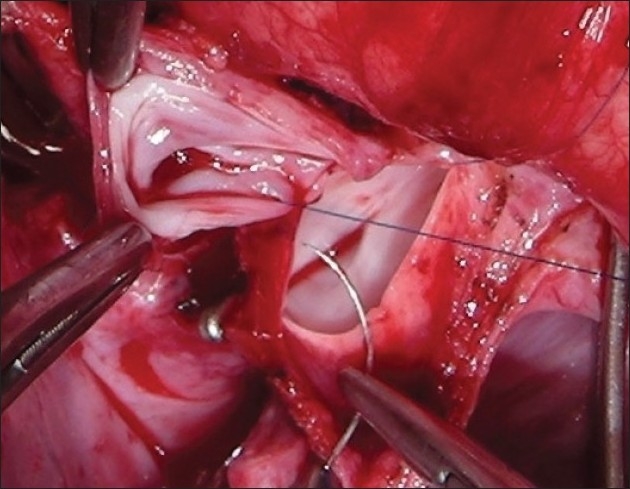
Superior vena cava to right pulmonary artery anastomosis in progress

**Figure 7:**
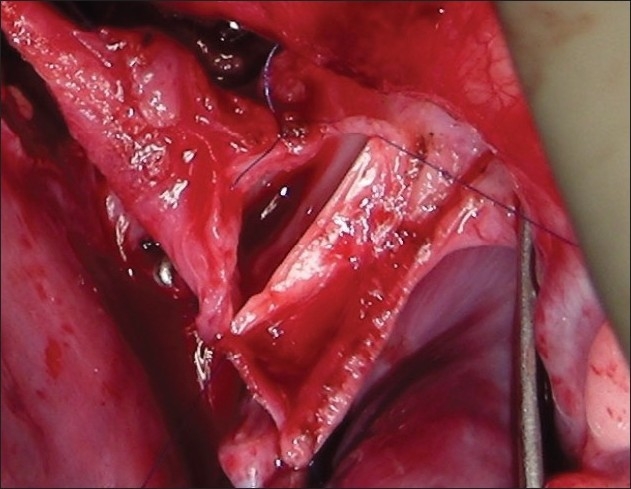
Anterior layer of Glenn shunt in progress

The procedure was performed under general anesthesia. The intra-operative management included monitoring electrocardiogram, (ECG), SaO_2_, EtCO_2_ and bispectral index (BIS) whenever possible.[11] Arterial blood gas (ABG) was analyzed at the baseline after intubation and during the procedure if the need be. However, the procedure time was usually short and therefore we rarely needed to do repeat ABG. In addition pressure monitoring line was placed in the in the SVC, an invasive arterial pressure line was placed in the radial or femoral artery and central venous access was obtained using a multiport cannula into either of the femoral veins. We electively started 5-7.5 mcg/kg/min of Dopamine before clamping the SVC and gave a volume load to the patient to elevate the mean arterial pressure so as to maintain adequate transcranial gradient (30-40 mm Hg). The naso-pharyngeal temperature was allowed to drift to 34 - 35° C. Heart was accessed through the midline sternotomy, pericardium opened and the cardiac anatomy assessed [Figure 1]. Since most of these patients were operated on the echocardiographic evaluation, the intra - operative pulmonary artery (PA) pressure was estimated in all the patients. The SVC was dissected completely and skeletonized from the junction with the innominate vein upto the cardiac end [Figure 2]. Thereafter, the right pulmonary artery (and left pulmonary artery if bilateral BDG shunt was to be done) was completely dissected from its origin to its division into the lobar branches [Figure 3]. In case of a functioning BT shunt, the distal end of the right pulmonary artery was not dissected before proceeding with the procedure. We electively clamped the pulmonary artery for upto 3-4 minutes and observed the changes in the oxygen saturation. Heparin was administered in a dose of 1 mg / kg body weight to achieve a desired activated clotting time (ACT) of 180 seconds or more. In addition, 30 mg / kg Methyl prednisolone was administered before dividing the SVC. After doing the test clamp on the PA, the right PA was opened on the superior aspect between stay sutures of 6/ 0 polypropylene. The control on the PA should be perfect, so as to ensure a bloodless field to achieve a good anastomosis [Figure 4]. The next step was to transect the SVC. An attempt was made to clamp the SVC below the azygous vein to allow some decompression of the SVC. Also, the SVC was clamped intermittently for two to three times before finally clamping it.. It was observed that the proximal SVC pressure settled down to lower values with each successive clamping [Figure 5]. The head end of the operating table was elevated to allow better venous drainage through the collateral pathways and the azygous vein. End to side anastomosis of the SVC to right PA was performed using 6 - O polypropylene [Figures 6 and 7]. The adequate flow through the BDG shunt was verified by observing the SVC pressure. Mean SVC pressure of 18-20 mm Hg was considered as being suggestive of a good flow through BDG. We electively started sodium nitroprosside infusion post-operatively to drop the left ventricular end diastolic pressure and the pulmonary artery mean pressure to ensure a proper functioning of the shunt.

**Figure 8:**
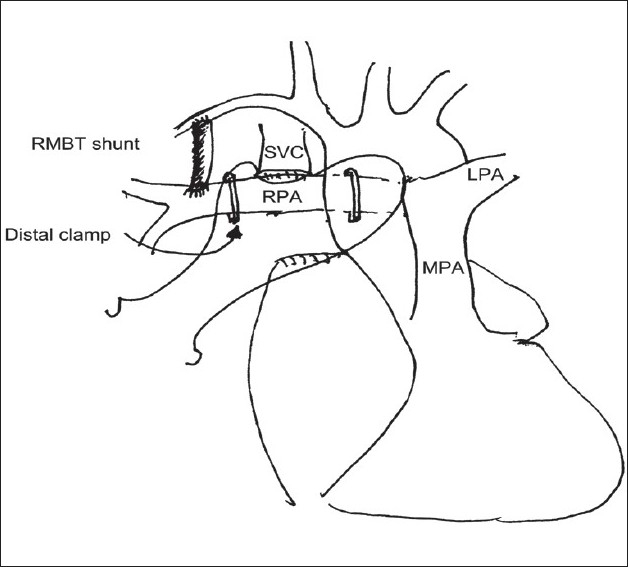
Placement of clamps during construction of Glenn anastomosis in patients with functioning right modified BT shunt. The distal clamp is placed proximal to the BT shunt. (Reproduced with permission Copyright © 2008 European Association for Cardio-thoracic Surgery)

**Figure 9:**
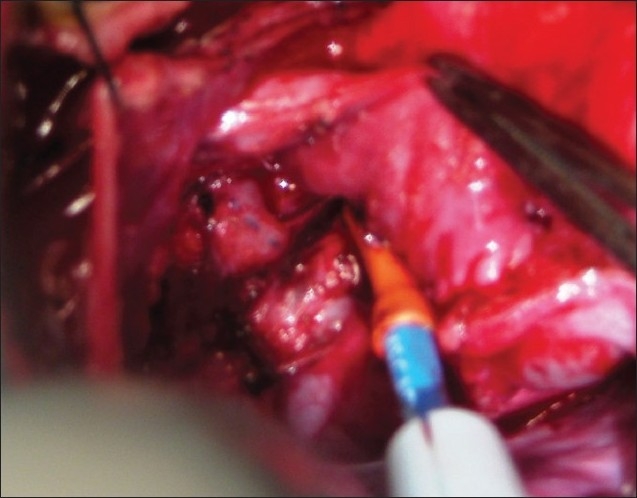
Right modified Blalock shunt dissected after completion of Glenn shunt

**Figure 10:**
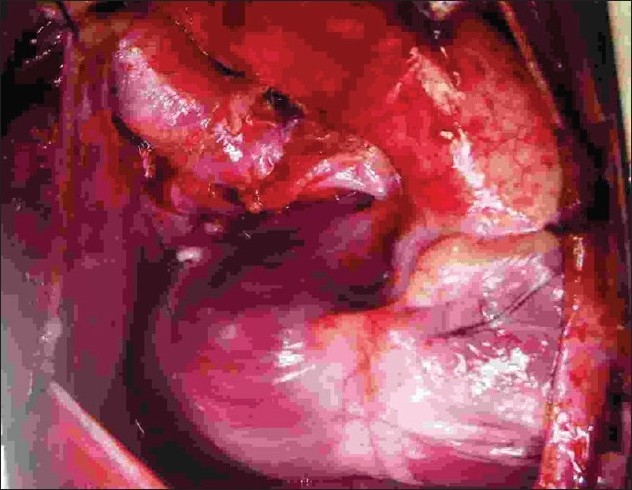
Completed procedure - Bidirectional Glenn shunt and division of right Blalock-Taussig shunt

**Figure 11:**
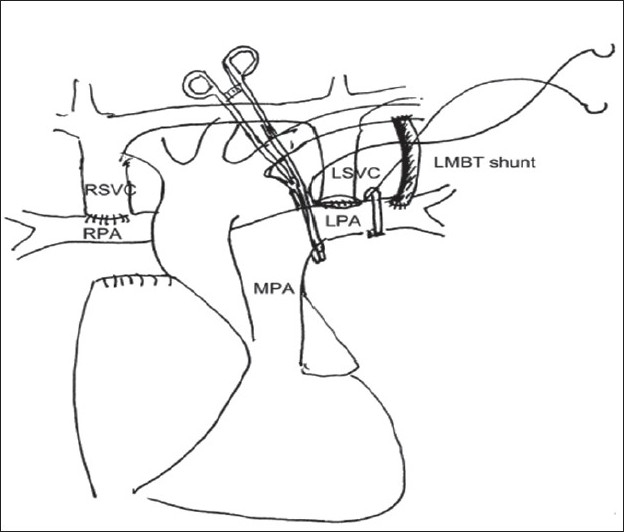
Construction of left Glenn anastomosis in a patient with functioning left modified BT shunt and bilateral superior vena cavae. The distal clamp is placed proximal to the BT shunt. The right Glenn anastomosis has already been completed earlier. (Reproduced with permission Copyright © 2008 European Association for Cardio-thoracic Surgery)

In presence of a functioning BT shunt, after ensuring the proper functioning of the BDG shunt, the BT shunt was dissected and divided or clipped [Figures 8, 9 and 10]. The systemic oxygen saturation, SVC pressure and mean arterial pressure was observed after the procedure. Azygous vein was either clipped or ligated at this stage. This was to ensure “no steal phenomenon” from the SVC to inferior vena cava through this vein. We did not use any drainage technique to decompress the proximal SVC. In small children, we used the Titanium clips (Ethicon Endo surgery™ LT 400, large) in place of the vascular clamps to temporarily occlude the right PA / left PA. This allowed more working space. Bilateral BDG shunt was performed with ease using the same technique [Figure 11]. In patients who needed to have bilateral BDG, the systemic venous hypertension was less of a problem, as one Glenn anastomosis was performed at one time and the other SVC was still draining. In patients who needed a bilateral BDG, we did the right BDG followed by the left BDG.[12] After the completeion of the anastomosis, the antegrade flow was preferably left open for better saturation and for preventing the development of arterio-venous malformations in the lungs. However, before taking this decision, the systemic arterial saturation and the venous pressure were taken into consideration; their detailed discussion is outside the scope of this review. If the antegrade flow was to be interrupted, the main PA was simply ligated.

Whenever feasible PA plasty was performed as a part of the procedure. This was done after the construction of the Glenn shunt. However, it is difficult to do it in small children. In bigger children, the right PA was usually clamped proximal to Glenn shunt to ensure adequate pulmonary blood flow and oxygenation before proceeding with the patching of the pulmonary arteries. Protamine was administered to reverse heparin at the end of the procedure.

### Post-operative care

Patients were stabilized in the ICU. After monitoring the SVC pressure for 3-4 hours, the internal jugular vein cannula was removed to prevent any jugular vein thrombosis. Patients were started on aspirin (5mk/Kg/day) which was continued indefinitely.

## RESULTS

There was no operative mortality in this group of patients. One patient needed a re-exploration for removal of clip on the left hemiazygous vein due to a missed diagnosis of IVC interruption. The increase in CVP on clamping the SVC ranged from 17-57 mm hg (mean 34.04 ± 10.15 mm hg). Mean SVC clamp time was 6.85 ± 1.52 min (range 4.5 - 10min). There was no haemodynamic instability during the procedure in any of the patients and the oxygen saturation was maintained at 65-70% throughout the procedure. Average duration of post-operative ventilation was 5.36 ± 3.12 hr. (range 2-24 hr). Mean ICU stay was 1.27 ± 0.45 days (range 1-2 days). There was no neurological event in the ICU in any of the patients. All the patients were discharged within one week of the procedure. Two patients developed pleural effusion that required drainage. All the patients had well functioning BDG shunt on the post operative echocardiogram.

As a part of the clinical study, the first 22 patients (operated at All India Institute of Medical Sciences, New Delhi) underwent a complete neurological evaluation, including the cognitive function assessment before discharge. The data was compared with the pre-operative data for any evidence of neurological deficit. These patients also had developmental assessment testing performed within one month of discharge. None of the patients had any deterioration of the DQ/IQ score in the post-operative period. A non contrast CT scan of the brain was done in all the patients pre and post-operatively. None of the patients had any evidence of hypoxic injury or venous infarcts.

None of the patients in this series needed conversion from off pump to on pump Glenn. We feel that proper patient selection and procedural planning helped in avoiding the use of CPB.

## CONCLUSION

BDG shunt (unilateral or bilateral) without CPB is a safe procedure in selected patients. It avoids CPB related problems and is economical, with excellent outcome. However, the procedure has to be planned properly to achieve the desired outcome.
